# Multi-Granularity Mask-Guided Network: An Integrated AI Framework for Region-Level Segmentation and Grading of Cataract Subtypes on AS-OCT Images

**DOI:** 10.3390/jcm15072798

**Published:** 2026-04-07

**Authors:** Yiwen Hu, Bingyan Hao, Yilin Sun, Yitian Zhao, Yuanyuan Gu, Fang Liu

**Affiliations:** 1 Department of Ophthalmology, Shanghai Tenth People’s Hospital, Tongji University School of Medicine, Shanghai 200072, China; 2 Ningbo Institute of Materials Technology and Engineering, Chinese Academy of Sciences, Ningbo 315000, China; 3 Ningbo Key Laboratory of Biomedical Imaging Probe Materials and Technology, Ningbo 315000, China

**Keywords:** artificial intelligence, cataract grading, anterior segment optical coherence tomography, multi-granularity feature extraction

## Abstract

**Objective**: To develop and validate an artificial intelligence (AI) system for automated lens opacities classification system III (LOCS III)-based grading of all three major cataract subtypes using anterior segment optical coherence tomography (AS-OCT). **Methods**: This is a single-center cross-sectional study. AS-OCT images were collected and manually graded by ophthalmologists according to LOCS III. The dataset was randomly split into training, validation, and test sets. We propose a novel multi-granularity mask-guided network (MMNet) that jointly performs lens substructure segmentation and severity grading. The model’s performance was assessed on an independent test set for automatic grading of cortical cataract (CC), nuclear cataract (NC), and posterior subcapsular cataract (PSC) and the grading performance of the proposed method against ophthalmologists was also evaluated. The model’s interpretability was assessed via attention heatmaps and feature visualization. **Results**: The proposed MMNet exhibited high agreement with ground truth conducted through gold standard. The proportions of predictions with an absolute error < 1.0 for three subtypes range from 83.02% to 89.94%. The model’s grading accuracy for cataract subtypes was between 82.20 ± 1.41% and 89.76 ± 1.31% among the three subtypes, the Area Under the Curve (AUC) was between 0.954 (95% CI, 0.952–0.969; *p* < 0.001) and 0.973 (95% CI, 0.964–0.985; *p* < 0.001). The MMNet shows a satisfactory mean absolute error (MAE) of 0.14 ± 0.35 in CC, 0.10 ± 0.30 in NC, and 0.17 ± 0.38 in PSC grading. It also achieved a fast grading speed of 0.0178 s/image against manual grading. **Conclusions**: The proposed AI model presented advanced performance on AS-OCT images in automated LOCS III-based cataract grading for CC and NC, and also showed feasibility in PSC assessment.

## 1. Introduction

Cataract, defined as lens opacity, remains the leading cause of reversible blindness globally, affecting over 95 million people and imposing a substantial socioeconomic burden that is exacerbated by the aging population [1]. This number will gradually increase over time, especially in low- and middle-income countries, where the number of people suffering from cataracts far exceeds the diagnosis and treatment capabilities of ophthalmologists. Cataract surgery is indicated when the progression of lens opacities leads to a clinically significant decline in visual function, thereby impacting the patient’s ability to perform daily activities. The impact on the patient’s vision remains the paramount consideration for surgical intervention. Accurate assessment of cataract severity is essential for making clinical decisions, surgical timing, and monitoring disease progression. The LOCS III is currently the most widely adopted clinical standard for cataract assessment [2]. However, the LOCS III relies heavily on subjective visual inspection and personal experience, making it vulnerable to inter- and intra-observer variability. In addition, its qualitative nature limits its ability to provide longitudinal, continuous, and objective monitoring of cataract progression. These limitations are especially problematic when evaluating the effects of non-surgical intervention, research or conducting precise follow-up assessments in early or slowly progressive cataract.

**Figure 1:**
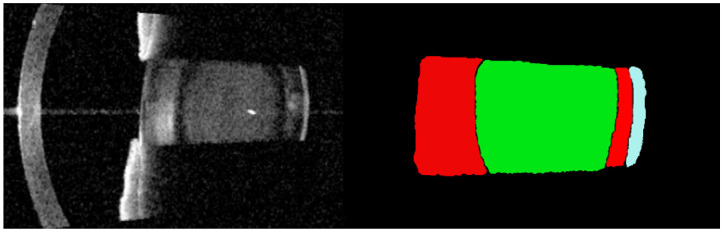
Demonstration of the anatomical localization of cataract. Cortical (red), nuclear (green) and posterior subcapsular (blue) cataracts, respectively.

AS-OCT has emerged as a promising objective imaging modality for cataract evaluation. A previous study has demonstrated strong agreement between AS-OCT-based measurements and LOCS III grading [3]. Slit-lamp photography, an examination machine that is widely used as a clinical standard, suffers from inter-observer variability and inadequate PSC assessment due to its two-dimensional projection nature. Unlike slit-lamp photography, AS-OCT images acquired from IOL-MASTER 700 (Carl Zeiss Meditec AG, Jena, Germany) can provide high-resolution, cross-sectional visualization of the crystalline lens with reduced dependence on lighting conditions, examiner skill, or image acquisition angle. Its ability to penetrate the lens, quantify optical density, and generate reproducible structural information makes it particularly suitable for follow-up and quantitative assessment (Figure 1). These advantages highlight AS-OCT imaging as a cutting-edge tool for objective cataract characterization [4,5,6,7].

Though they exhibit several potentials in cataract assessment, current examination apparatuses do not provide automated subtype segmentation and grading. To identify the cortical, nuclear, and posterior subcapsular regions, they still require manual labeling and expert grading, which is time-consuming, labor-intensive, and impractical for large-scale clinical deployment. To address this challenge, AI models offer a natural solution [8]. Recent studies have explored various AI-based grading methods using different imaging modalities, including slit-lamp photography, fundus photography, and optical coherence tomography [9,10,11,12]. Deep learning has achieved remarkable success in medical image analysis, with convolutional neural networks (CNNs) playing a foundational role in tasks such as classification, segmentation, and detection. In recent years, transformer-based architectures and hybrid models have further advanced the field by capturing long-range dependencies and multi-scale representations [13,14,15]. However, these models often require large-scale datasets and substantial computational resources, which may limit their applicability in specialized medical imaging tasks with relatively small sample sizes. Moreover, their global attention mechanism, while powerful for capturing long-range dependencies, may sometimes overlook fine-grained local details that are critical for precise boundary delineation. This is a key consideration in tasks like lens subregion segmentation in AS-OCT images. Hybrid models attempt to balance these trade-offs, but their design often prioritizes general-purpose performance over anatomical priors. Several studies have investigated AI-based cataract assessment using different imaging modalities and deep learning strategies. A comparison of representative studies is summarized in Table 1. As shown in Table 1, previous studies mainly focus on either segmentation or grading tasks and often use or develop a series of architectures to conduct the study. However, the automated, simultaneous segmentation and grading of all three major cataract subtypes on AS-OCT images remain a challenge, especially the objective evaluation of PSCs, a subtype most likely to cause rapid vision loss.

To address this gap, we proposed a multi-granularity mask-guided network that is capable of rapid and precise full-region segmentation as well as LOCS III-based grading of CC, NC, and PSC on AS-OCT images. This AI framework was designed to address the limitations of subjective visual assessment, enable quantitative monitoring, and provide an assistant tool for both clinical practice and future research. A preliminary version of this work was presented at the IEEE International Symposium on Biomedical Imaging (ISBI) 2026 and is currently in press. That version focused on the core methodological innovations. The present manuscript substantially extends it with comprehensive clinical validation, systematic comparison with modern architectures, enhanced interpretability analysis, and expanded discussion of clinical implications.

## 2. Materials and Methods

### 2.1. Data Collection

This prospective, single-center, cross-sectional study enrolled eligible patients during clinical visits and preoperative consultations for cataract surgery between December 2021 and May 2025. All participating patients provided informed consent before they were included in the dataset. AS-OCT images were obtained from the ophthalmology database of Shanghai Tenth People’s Hospital Affiliated to Tongji University. Each patient was imaged once at a single time point and no repeated AS-OCT imaging over and no longitudinal follow-up data were collected. All images were acquired by a single operator trained in a standardized protocol. All participants underwent bilateral imaging using the IOL-MASTER 700, with standardized head positioning and consistent ambient illumination maintained throughout all imaging sessions to ensure data uniformity.

The inclusion criteria for this study were: (1) age above 40 years; (2) diagnosis of cataracts according to the LOCS III; and (3) provision of signed informed consent by the patient. Exclusion criteria comprised (1) patients with a history of ocular surgery, ocular trauma, or ocular diseases other than cataract; and (2) eyes with inadequate lens visualization due to poor fixation or dislocation, precluding reliable cataract assessment.

A total of 2117 eyes from 1682 patients were ultimately included in this study. The dataset was randomly split at the patient level into three mutually exclusive subsets: training, validation, and test sets. Specifically, 1527 eyes from 1125 patients were assigned to the training set, 272 eyes from 258 patients to the validation set, and 318 eyes from 299 patients to the test set. Each patient was assigned to only one subset to prevent data leakage and minimize overfitting.

### 2.2. Cataract Labeling

Cataracts were graded using AS-OCT images acquired from IOL-MASTER 700 (Carl Zeiss Meditec AG, Jena, Germany), in strict accordance with LOCS III. NCs were graded on an integer scale from 1 to 6, while CCs and PSCs were assessed from 1 to 5 according to photographic transparency standards. Two ophthalmologists with over 10 years of clinical experience in cataract management independently graded each image. When the two graders disagreed, a third cataract specialist with over 20 years of clinical experience adjudicated the case. The final consensus grade was used as ground truth. For the segmentation task, the model was trained to delineate lens structures in AS-OCT images from the IOL-MASTER 700. Training labels were manually annotated, with each AS-OCT image segmented into three distinct regions: cortical, nuclear, and posterior subcapsular. During the initial annotation phase, a subset of 200 images was independently annotated by two senior ophthalmologists with over 20 years of experience. The inter-rater agreement was quantified using Dice coefficients, yielding values of 0.933 ± 0.029 for the cortical region, 0.948 ± 0.026 for the nuclear region, and 0.849 ± 0.037 for the posterior subcapsular region. Given the observed inter-rater agreement with limited variability between ophthalmologists, the remaining annotations were completed by one senior ophthalmologist to ensure consistency across the dataset, and only the annotations generated by this ophthalmologist were used for the model.

### 2.3. Artificial Intelligence Modeling and Implementation Details

**Figure 2:**
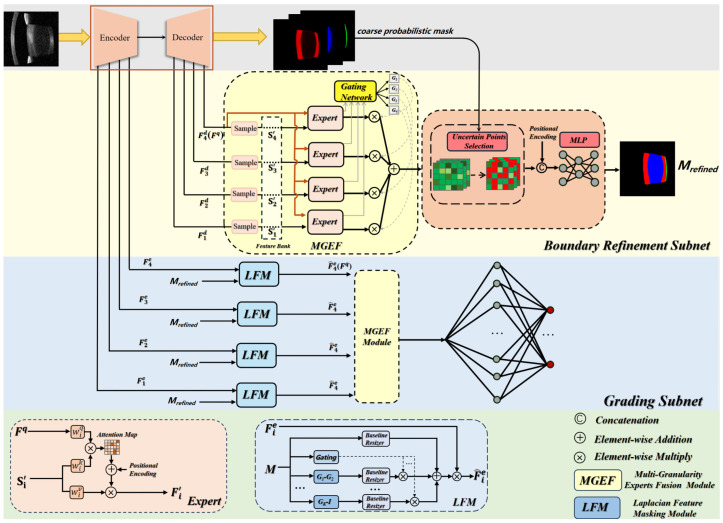
Overall workflow of the proposed MMNet. The framework adopts an encoder–decoder backbone for initial segmentation, followed sequentially by the boundary refinement subnet and the grading subnet. The boundary refinement subnet produces fine-grained masks, which serve as spatial priors to guide the grading subnet. Finally, three parallel grading subnets are used to simultaneously perform severity grading of cortical, nuclear, and posterior subcapsular cataracts.

The MMNet framework was designed to segment the lens into cortical, nuclear, and posterior subcapsular regions (Figure 2). The model adopted a U-Net as its backbone, consisting of an encoder that progressively downsamples the input and a decoder that recovers spatial details through skip connections [19]. Three specialized modules are integrated with the backbone: Multi-Granularity Expert Fusion (MGEF), Implicit Boundary Rendering (IBR), and Laplacian Feature Masking (LFM). The MGEF adaptively integrated features from multiple semantic levels to address scale variations among lens subregions. IBR refined boundary predictions using an implicit neural representation with uncertainty-guided point sampling. LFM isolated subregion-specific features via frequency-aware mask refinement, enabling clean feature separation for grading. A detailed architecture description is provided in Appendix A.

The MMNet model was implemented in PyTorch (version 2.7.1; Meta Platforms, Inc., Menlo Park, CA, USA) with a U-Net architecture as the backbone. All input images were standardized to a resolution of 224 × 224 pixels. The network was trained using the AdamW optimizer with an initial learning rate of 5 × 10^−3^ and a polynomial learning rate decay scheduler. The batch size was set to 16. The network was optimized using a weighted combination of cross-entropy loss for the grading task and Dice loss for the segmentation task. The model was trained for 200 epochs with early stopping based on validation loss. All experiments were conducted on a workstation equipped with an NVIDIA GeForce RTX 3090 GPU. To enhance data diversity and robustness, data augmentation was applied during training, including random vertical flipping and random rotation within ±15°. These augmentations were applied exclusively to the minority-class samples in the training set to mitigate class imbalance.

### 2.4. Model Visualization and Interpretability

To evaluate the interpretability of the proposed model, several complementary visualization techniques were applied. To provide baseline comparisons for the learned feature representations, two widely used deep learning architectures, ConvNeXt and Vision Transformer (ViT), were implemented as benchmark models [20,21]. ConvNeXt is a modernized architecture that has demonstrated great performance across various image recognition tasks, while ViT represents a transformer-based architecture that models global dependencies through self-attention mechanisms. Both benchmark models were trained using the same training, validation, and test splits as MMNet, with identical preprocessing and optimization settings. To assess the classification behavior of MMNet, confusion matrices were generated for each cataract subtype on the test set. The matrices compare the model’s predicted grades with ground truth labels, where diagonal elements indicate correct classifications and off-diagonal elements represent misclassification patterns. The extracted feature embeddings from the models were visualized using t-Distributed Stochastic Neighbor Embedding (t-SNE) for qualitative comparison. Heatmaps were generated to visualize the spatial regions contributing to the model’s grading decisions.

### 2.5. Evaluation Metrics and Statistical Analysis

SPSS software (version 26.0; SPSS, Inc., Chicago, IL, USA) and R software (version 4.5.1; R, Inc., Vienna, Austria) were used for analysis. To evaluate the segmentation results, we employ Pixel Accuracy (PA), Intersection-over-Union (IoU), and Hausdorff Distance (HD) as metrics to measure the accuracy of the auto-segmented area. To assess the grading performance, the distributions of the differences between the predicted grading values and the true values are presented. The percentage of predictions within ±1.0 grade of the ground truth was reported using the following formula: $Rel.0=\frac{1}{N}\sum_{i=1}^{N}I\left(\left|{\tilde{y}}_{i}-y_{i}\right|<1.0\right)$, where *N* denotes the total number of AS-OCT images; ${\tilde{y}}_{i}$ represents the predicted cataract grade; $y_{i}$ is the corresponding ground truth grade; and I(x) is an indicator function that returns to one if x is true or zero otherwise. To comprehensively evaluate the performance of the proposed method, we adopted widely accepted classification metrics for cataract grading based on AS-OCT images, including accuracy (ACC), precision (PRE), sensitivity (SEN), specificity (SPE), and F1 score. These metrics are defined as follows: ACC = (TP + TN)/(TP + TN + FP + FN), PRE = TP/(TP + FP), SEN = TP/(TP + FN), SPE = TN/(TN + FP), and F1 = 2(PRE × SEN)/(PRE + SEN). Here TP, FP, TN, and FN are true positive, false positive, true negative, and false negative, respectively. The performance metrics are reported as the mean ± standard deviation obtained from five-fold cross-validation. Inter-rater agreement between the model and the reference standard was quantified using Cohen’s quadratic-weighted kappa (QWK), which accounts for the ordered nature of LOCS III grades. Receiver operative characteristic (ROC) curves were created using the one-vs.-rest approach for each cataract subtype, and the areas under the curve were computed using the pROC package (version 1.18.5) in R. Macro-averaging was applied to obtain a single AUC value per subtype, summarizing overall discriminative performance across all severity grades. Statistical comparisons of AUC values were performed using the DeLong test [22].

Before further analysis, normality and homogeneity of variance were verified. For continuous variables, one-way analysis of variance (ANOVA) was used to compare differences among groups when data were normally distributed and variances were homogeneous. Otherwise, the Kruskal–Wallis H test was applied for multi-group comparisons and the Mann–Whitney U test for pairwise post hoc or two-group analyses when appropriate. For a categorical variable, the Chi-square test was used to compare distributions across groups. Interclass correlation coefficients (ICC_inter_) of the two grading ophthalmologists were calculated using a two-way mixed-effects model with absolute agreement. Mean predicted grades were calculated for all raters (AI model and one experienced ophthalmologist other than those mentioned in the Cataract Labeling section) and compared against the ground truth reference labels. Intra-observer variability was assessed by having the senior ophthalmologist grade the test set three times, with intervals of one week and two weeks between sessions. The final reference grade was defined as the mode of the three repeated gradings. Intraclass correlation coefficients (ICC_intra_) (two-way mixed and absolute agreement) were then calculated. Grading consistency was assessed by computing the mean absolute error and mean squared error (MSE) between each rater’s grade and the ground truth. A *p* value of less than 0.05 was considered statistically significant.

## 3. Results

Originally, 3412 eyes were included in the present research. According to the exclusion criteria, 2117 eyes of AS-OCT images were included in the final dataset. These were randomly separated into a training set (1527 eyes), a validation set (272 eyes), and an independent test set (318 eyes). A summary of the dataset distribution is provided in Table 2. The age distribution of the dataset is provided in the Supplementary Materials (Table S1). The ICC_inter_ values of the two grading ophthalmologists were 0.885 (95% CI, 0.876–0.894; *p* < 0.001), 0.946 (95% CI, 0.939–0.952; *p* < 0.001), and 0.840 (95% CI, 0.786–0.876; *p* < 0.001) for CCs, NCs, and PSCs, respectively. Demographic and clinical characteristics are presented in Table 3. No statistical significance was observed in age or sex among the three subsets. For cataract severity grades, CC showed no significant differences among the three subsets (*p* = 0.27). However, statistically significant differences were found for NC (*p* = 0.01) and PSC (*p* < 0.001). Specifically, the test set had a higher mean grade for NC (3.37 ± 1.64) compared to the training (3.14 ± 1.57) and validation (2.97 ± 1.53) sets, and a higher mean grade for PSC (2.33 ± 1.51) compared to the training (1.64 ± 1.03) and validation (1.61 ± 1.02) sets.

The segmentation results for the cortical, nuclear, and posterior subcapsular regions are shown in Table 4. The MMNet achieved exceptional PA and IoU across all cataract subtypes. In addition, the model obtained IoUs of 85.42 ± 1.03% for CC, 91.98 ± 0.92% for NC, and 60.84 ± 2.14% for PSC. The HD remained low for all three regions, indicating precise boundary localization.

The grading performance of the MMNet for CCs, NCs, and PSCs on the test dataset is summarized in Table 4. The MMNet exhibited consistent grading accuracy across all three cataract subtypes. The proportions of absolute grading error < 1.0 were 85.53% for cortical cataract, 89.94% for nuclear cataract, and 83.02% for PSCs.

Across all subtypes, the MMNet achieved sensitivity ranging from 81.13 ± 1.25% to 89.86 ± 1.21%, specificity from 81.94 ± 1.13% to 89.29 ± 1.14%, and overall accuracy from 82.20 ± 1.41% to 89.76 ± 1.31%. The model also reported precision from 79.57 ± 1.17% to 88.39 ± 1.29%, and F1 scores from 79.49 ± 1.53% to 89.27 ± 1.38%, reflecting performance across different severity levels for all subtypes. The performance of the MMNet in NCs was the highest, while PSCs showed relatively lower metrics.

**Figure 3:**
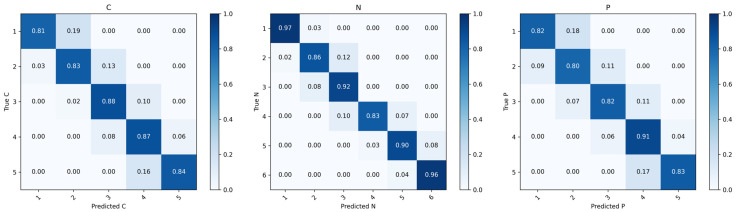
Confusion matrices for grading of CCs, NCs, and PSCs. From **left** to **right**, the matrices correspond to CCs, NCs, and PSCs, respectively. The y-axis indicates true grades and the x-axis indicates predicted grades. The diagonal elements correspond to correct classifications.

The confusion matrices for CCs, NCs, and PSCs are shown in Figure 3. For CC, correct classification rates ranged from 0.81 to 0.88 across grades. Misclassifications were mainly observed between adjacent grades, particularly between grades 1 and 2, as well as grades 4 and 5. NC showed stability, with correct classification rates between 0.83 and 0.97. Misclassifications were predominantly limited to neighboring grades. For PSC, correct classification rates ranged from 0.80 to 0.91. Most misclassifications occurred between adjacent severity levels, such as predicted grade 5 and grade 4. No substantial misclassification across non-adjacent grades was observed.

**Figure 4:**
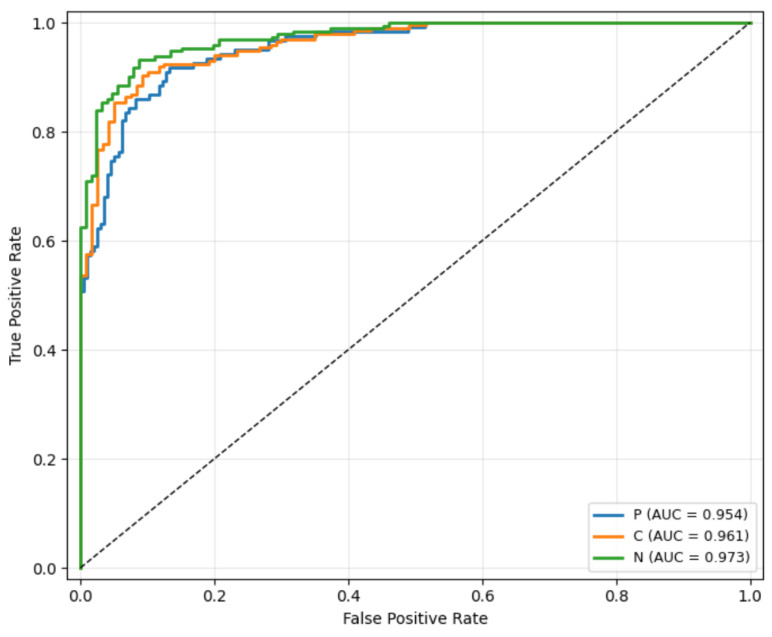
Receiver operating characteristic curves for global grading performance. Global multi-class ROC curves summarizing overall classification performance for CCs, NCs, and PSCs. The diagonal dashed line indicates the performance of a random classifier.

The global multi-class ROC curves for each cataract subtype are presented in Figure 4. The MMNet achieved an overall AUC of 0.954 (95% CI, 0.952–0.969; *p* < 0.001) for PSC, 0.973 (95% CI, 0.964–0.985; *p* < 0.001) for NC, and 0.961 (95% CI, 0.959–0.978; *p* < 0.001) for CC. To assess concordance between automated and manual grading, QWKs were calculated for each subtype (Table 4). The QWK values were 0.87 for CCs, 0.95 for NCs, and 0.89 for PSCs, showing agreement between the MMNet and ground truth.

To further assess the performance of the proposed model, we compared the MMNet with two representative deep learning architectures, ConvNeXt and ViT. As shown in Table 5, the MMNet achieved higher accuracy, sensitivity, and F1 score than the benchmark models. ConvNeXt demonstrated competitive performance but remained inferior to MMNet in these metrics. A similar pattern was observed for the ViT model. In terms of specificity, the MMNet achieved 83.52 ± 1.08% for CC, 89.29 ± 1.14% for NC, and 81.94 ± 1.13% for PSC. ConvNeXt and ViT showed higher specificity values, particularly for NC. Overall, MMNet maintained stronger performance across the evaluated metrics.

To assess the clinical relevance of the MMNet, we compared its grading performance with that of an independent senior ophthalmologist who was not involved in the ground truth labeling process (Table 6). The ICC_intra_ values of the doctor were 0.864 (95% CI, 0.839–0.886; *p* < 0.001) for CC, 0.936 (95% CI, 0.923–0.947; *p* < 0.001) for NC, and 0.825 (95% CI, 0.794–0.853; *p* < 0.001) for PSC. For CC, the mean grade assigned by the MMNet (2.88 ± 1.02) was similar to those of the senior doctor (2.87 ± 1.01), with no significant difference relative to the ground truth (*p* = 0.65). A similar trend was observed in NC, where the MMNet yielded a mean grade of 3.39 ± 1.64, closely matching clinician performance and showing no statistical difference versus the ground truth (*p* = 0.97). For PSC, the MMNet produced a mean grade of 2.40 ± 1.45, again comparable to ophthalmologists and consistent with the ground truth (*p* = 0.81). Regarding absolute grading error, the MMNet achieved MAE values of 0.14 ± 0.35 for CC, 0.10 ± 0.30 for NC, and 0.17 ± 0.38 for PSC. The MAEs for the senior doctor were higher in CC (*p* = 0.04) and in PSC (*p* = 0.01) than those for the MMNet. Mean squared error showed a similar pattern. The MMNet demonstrated potential advantages in grading efficiency compared with doctors, requiring an average of 0.0178 s per image versus 8.89 s for senior doctors. This nearly three orders of magnitude acceleration highlights the computational efficiency of the MMNet and suggests that automated grading may facilitate rapid cataract assessment in high-volume clinical settings.

**Figure 5:**
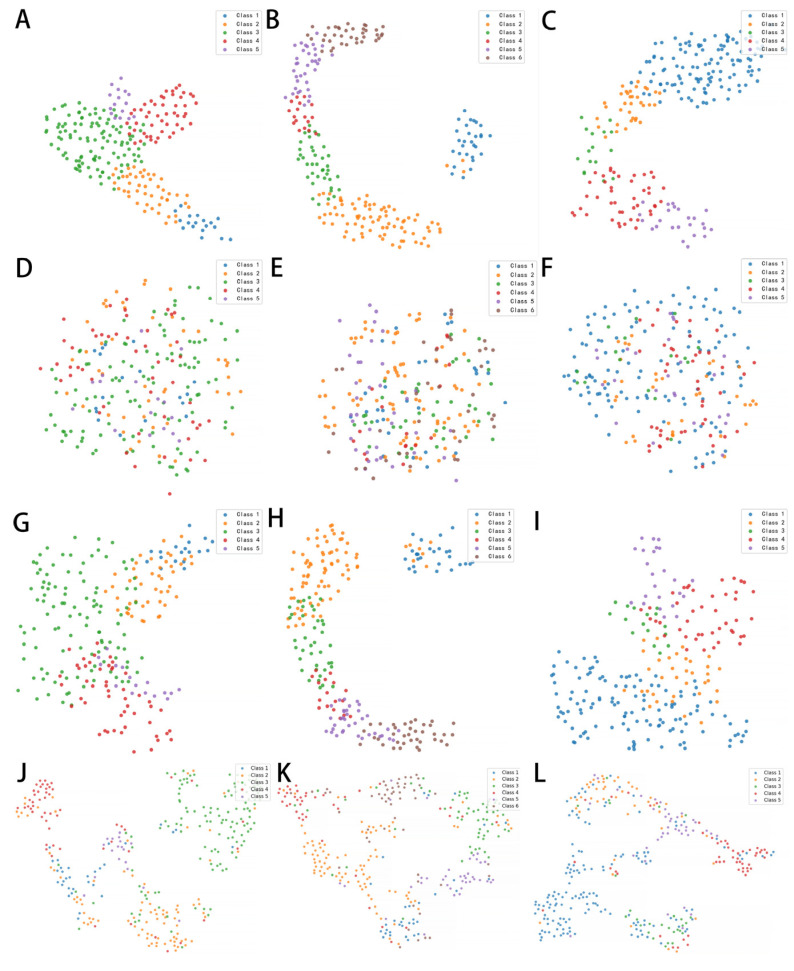
The t-SNE visualization of learned features by different methods. (**A**–**C**) showcase the image features extracted from MMNet. (**D**–**F**) depict the original image features, revealing a dispersed distribution. (**G**–**I**) showcase the features derived from the traditional CNN ConvNeXt. (**J**–**L**) exhibit the features derived from transformer-based network Vision Transformer.

The t-SNE visualization of learned feature embeddings is shown in Figure 5. Figure 5A–C illustrate the feature distributions extracted by the MMNet for CCs, NCs, and PSCs, respectively. Compared with raw image features and ConvNeXt representations, the embeddings produced by the MMNet appear to show relatively more organized grouping patterns across severity grades. In contrast, the raw image features (Figure 5D–F) exhibited scattered and overlapping distributions with no discernible clustering pattern. ConvNeXt features showed only partial grouping of severity levels, with greater overlap observed between adjacent grades in the t-SNE visualization (Figure 5G–I). A similar pattern was also observed in ViT from the t-SNE visualization (Figure 5J–L).

**Figure 6:**
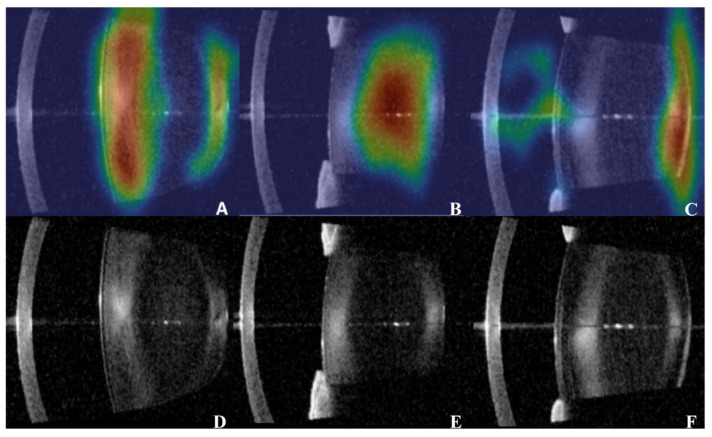
Heatmaps of attention recognition by MMNet for different segments of AS-OCT images. (**A**) The part of interest when the model identifies cortical regions. (**B**) The part of interest when the model identifies nuclear regions. (**C**) The part of interest when the model identifies posterior subcapsular regions. (**D**–**F**) The original images for heatmap (**A**) to heatmap (**C**), respectively.

The attention heatmaps generated by the MMNet for different cataract subtypes are presented in Figure 6. When the MMNet identifies CC (Figure 6A), the model focuses on the peripheral spoke-like opacities consistent with clinical inspection. For NC (Figure 6B), attention was concentrated on the central lens nucleus, corresponding to the expected anatomical distribution of nuclear sclerosis. For PSCs (Figure 6C), the model highlighted the posterior capsular region where plaques typically develop.

## 4. Discussion

In the present study, we have developed the MMNet, which is capable of simultaneously performing lens substructure segmentation and LOCS III-based severity grading for CCs, NCs, and PSCs using AS-OCT images. This model is designed to achieve full-region, three-subtype, and clinically standardized grading in an integrated pipeline using AS-OCT, addressing several long-standing challenges in both cataract imaging and AI-assisted ophthalmic diagnostics. Across all major evaluation metrics, MMNet exhibited strong agreement with clinician assessments and high computational efficiency, highlighting its potential role as a decision support tool for objective cataract quantification.

Early and accurate cataract grading weighs a lot in decision-making in both surgical planning and longitudinal postoperative management. While LOCS III remains the globally accepted standard, its performance is inherently constrained by subjectivity, variations in illumination, and observer experience. This variability is amplified in community or primary care settings, where direct access to skilled ophthalmologists is limited. The need for objective, reproducible, and scalable cataract assessment tools has therefore become increasingly urgent, especially given the rising global burden of early onset cataracts driven by high myopia, metabolic diseases, corticosteroid exposure, and lifestyle factors [23,24,25]. AI-assisted systems present a promising avenue for mitigating disparities in access and improving diagnostic accuracy across different levels of care.

AS-OCT enables high-resolution visualization of the entire lens structure from the anterior cortex to the posterior capsule. This imaging modality provides clear, structurally consistent, and artifact-reduced cross-sectional images of ocular tissues. A key advantage of AS-OCT is its deeper tissue penetration capability, which allows for detailed assessment of PSCs that may be obscured in other imaging techniques [26]. Wang et al. previously reported good agreement between the outcomes from AS-OCT images and from conventional slit-lamp images [3]. This finding suggests that the present modality may serve as a reliable basis for objective quantification. In our work, AS-OCT enabled robust feature extraction across all cataract subtypes. The PSCs which often exhibited low contrast and subtle boundaries under slit-lamp imaging were also better extracted from AS-OCT images. As shown in Table 2, the dataset used in this study exhibits a class imbalance in the severity grading distribution, particularly for PSC, where grade 1 and grade 2 are predominant. This real-world data characteristic often leads to degraded model performance on minority classes. As shown in Table 3, significant differences were observed in NC (*p* = 0.01) and PSC (*p* < 0.001) severity distributions across the training, validation, and test sets, whereas CC grades remained balanced (*p* = 0.27). These differences likely reflect the inherent characteristics of cataract subtypes. NC spans a broader severity spectrum and is therefore more susceptible to sampling variation, whereas PSC is relatively rare and skewed toward lower grades, making distributional imbalance more likely during random splitting even at the patient level. Given that each AS-OCT image contains simultaneous grades for CC, NC, and PSC, conventional single-label stratified sampling is not readily applicable in this setting. Nevertheless, such distribution differences are unlikely to introduce substantial evaluation bias. The model exhibited generally consistent performance across multiple metrics, including accuracy, F1 score, MAE, and QWK, and showed stable performance across different severity levels. Notably, the relatively higher PSC grades in the test set may provide a more stringent assessment of model performance in challenging cases.

The metrics of comprehensive performance in Table 4 indicate that this imbalance did not cause severe degradation or bias in the model’s performance. The F1 scores across all three subtypes (83.66% for CC, 89.27% for NC, and 79.49% for PSC) remained at a high level; this is particularly noteworthy because the F1 score, the harmonic mean of precision and recall, is highly sensitive to class imbalance. Although the F1 score for PSC is slightly lower, it still approaches 80% and indicates that the model achieves reliable overall classification performance under this data distribution. More importantly, the model shows a better-balanced performance between SEN and SPE. Sensitivity (81.13%) and specificity (81.94%) for PSC are nearly equal. This suggests that the model does not simply “cheat” by predicting all samples as the majority class to inflate overall accuracy. It genuinely strives to distinguish between cases of different severity levels. This balance underscores the reliability of our model’s performance on imbalanced data. Another factor that may influence model performance is the inherent variability in manual LOCS III grading, which serves as the ground truth. In the present study, two experienced ophthalmologists independently graded all images, and their ICC_inter_ values were 0.885 for CCs, 0.946 for NCs, and 0.840 for PSCs. These values show excellent agreement for NC and good agreement for cortical and PSC subtypes and support the reliability of the reference standard. However, the relatively lower ICC_inter_ value for PSC may infer greater subjectivity in grading PSCs, which may partly explain the lower model performance observed for this subtype. Despite this variability, the model achieved QWK values with ground truths of 0.87, 0.95, and 0.89 for cortical cataract, nuclear cataract, and PSC, respectively. These values are comparable to the inter-rater agreement among human doctors. This finding suggests that the MMNet may learn consistent patterns from the training data and potentially reduce the impact of individual grader variability.

The MMNet integrates multi-granularity feature learning with anatomical priors to support both segmentation and severity grading. The model achieved high accuracy and interobserver agreement in CC and NC subtypes, especially with the strongest performance in nuclear grading (Table 4). This is quite important because NC strongly correlates with phacoemulsification energy and postoperative endothelial cell loss [27]. The high QWK observed for the MMNet suggests that the model may support objective preoperative assessment of cataract severity. The MAE and MSE further reflected the reliability of the MMNet’s grading performance, as they quantify the numerical deviation between the predicted and ground truth LOCS III grades, as shown in Table 6. Though the senior doctor presented better grading capability in CC and PSC with statistical significance, these values of the MMNet were still low and close to the senior doctor’s performance, especially in NC grading. MSE values also remained low across all subtypes, suggesting no large outlier errors. From these findings, we may infer that the prediction of the MMNet typically deviated from the ground truth by less than one grade, which is unlikely to alter clinical decision-making in the majority of cases.

PSC grading remains particularly challenging. PSC lesions are typically small and located near the posterior capsule, often presenting with relatively low contrast on slit-lamp photography. In the present study, the MMNet exhibited feasibility in PSC grading, with an AUC of 0.954, and balanced sensitivity and specificity in the test dataset. However, we have noticed that the outcomes should be interpreted in the context of class imbalance among the PSC grades. As shown in Table 4 and Table 6, MMNet yielded a lower F1 score of 79.49% in PSC and higher MAE compared to CC and NC. The confusion matrix of PSC further revealed that while grade 4 PSC achieved the highest correct classification rate of 0.91, grade 5 showed a correct classification rate of 0.83, with 0.17 of cases misclassified as grade 4 (Figure 3). This pattern of misclassifications confined to adjacent grades may indicate that the model captures the ordinal nature of cataract severity, even for higher-grade PSC lesions. Nevertheless, the relatively lower performance on grade 5 PSC is likely to reflect the limited number of severe PSC cases in the training set (41 cases of grade 5 in the training dataset versus 41 in the test dataset), which may hinder the model’s ability to learn distinctive features for advanced PSC. Segmentation analysis further highlights the intrinsic challenges of PSC assessment. Though the MMNet achieves preferable performance in CC and NC subregions, there is still room for improvement in the challenging segmentation of PSC [28]. In this area, AS-OCT images are often affected by reflection, shadowing, and partial volume effects. The posterior capsule appears as a thin, hyper-reflective line and the boundary between the capsule and PSC lesions is often indistinct, which complicates precise annotation and segmentation.

In the benchmark comparison, ConvNeXt and ViT show higher specificity in certain cataract subtypes, particularly NC (Table 5). This pattern suggests that these architectures tend to produce more conservative predictions, reducing false-positive classifications but potentially missing subtle cases, which may explain their relatively lower sensitivity and F1 scores. In contrast, the MMNet achieves a more balanced performance across sensitivity and specificity, which may reflect its improved capability in distinguishing adjacent cataract severity grades. Recent hybrid architectures such as ViT-Adapter and Swin-UNet have demonstrated the advantages of combining convolutional and transformer-based feature extraction for medical image segmentation [29,30]. These models provide strong global context modeling but often require larger datasets and greater computational resources. In contrast, the MMNet incorporates anatomically grounded modules (the LFM module is described in Appendix A) that explicitly encode clinical priors about lens substructures, improving efficiency and interpretability for cataract grading. In addition, the use of implicit neural representations and high-frequency positional encoding enables the MMNet to capture subtle structural variations that may be attenuated in conventional architectures. Despite this advantage, segmentation performances differ across cataract subtypes. PSC shows the lowest segmentation accuracy and also the weakest grading performance, whereas CC and NC achieve higher segmentation accuracies (IoU > 85%) and better grading metrics. As the grading module in our framework is guided by segmentation masks that serve as spatial priors for feature extraction, the consistency between segmentation accuracy and grading performance further supports the effectiveness of the mask-guided design. However, other factors such as class imbalance, small lesion size, and grading subjectivity may also contribute to the observed differences. Interpretability is also critical in the use of medical artificial intelligence. T-SNE analysis has revealed that the MMNet generated feature embeddings with clearer inter-grade separation compared with raw image features and those extracted by ConvNeXt and ViT (Figure 5). Noticeable overlap between adjacent grades remains in the ConvNeXt and ViT feature spaces, especially for CCs and PSCs. This observation is consistent with the quantitative results, where both ConvNeXt and ViT achieve lower accuracy and F1 scores compared with the MMNet, despite showing relatively high specificity in some categories. These observations indicate that MMNet captures disease-relevant structural variations more effectively than conventional CNN and transformer-based architectures.

The attention heatmaps (Figure 6) provide visual explanations into the model’s decision-making process. As the model achieved high IoUs of 85.42% and 91.98% for CC and NC, respectively, the heatmaps reveal a consistent focus on the peripheral cortex and central nucleus. These areas are those that ophthalmologists routinely assess. In contrast, despite that a lower IoU of 60.84% for PSC is reported, the heatmap still highlights the posterior capsule region most (Figure 6C). This finding implies that the model can still identify the correct anatomical location. The observation is consistent with the segmentation and grading performance patterns described earlier. The heatmaps illustrate that the MMNet consistently focuses on the anatomical regions relevant to each cataract subtype. These regions correspond precisely to the areas ophthalmologists evaluate during clinical examinations. Such biologically plausible attention patterns can contribute to the transparency and clinical trustworthiness of the system.

The findings of this study reveal potential clinical utility. Automated, region-level cataract assessment may improve consistency in severity grading, reduce reliance on subjective evaluation, and enable standardized monitoring of progression. While AS-OCT equipment is more expensive than conventional slit-lamp examination, the rapid grading speed of MMNet (0.0178 s/image) and its interpretability via attention heatmaps suggest that the model could support integration into high-volume cataract clinics, remote screening programs, and resource-limited primary healthcare settings where access to specialists is limited. The potential for automated, objective grading and reduced reliance on specialist time may offer long-term value in such settings. It should also be noted that while cataract grading is important for ophthalmologists in surgical decision-making, a comprehensive assessment of multiple clinical factors is required, and the final judgment remains with the surgeon. Therefore, the current model is intended to provide objective grading as a decision support tool. Additionally, AS-OCT-based automated grading could also support research applications requiring quantitative endpoints, for instance, pharmacologic interventions for cataract or longitudinal natural studies in high-risk populations [31].

Despite the promising results, several limitations of this study should be acknowledged. Firstly, this study was based on a single-center dataset, which may lack ethnic and demographic diversity. Consequently, the generalizability of our findings to other populations with different patient demographics and clinical practices remains uncertain. External validation using multi-center or internationally diverse datasets is essential to further establish the robustness and generalizability of the model across varied populations. Secondly, the MMNet was trained and validated on images from a single device (IOL-Master 700). Device-specific imaging characteristics, such as differences in resolution, signal intensity, and noise distribution, may introduce domain bias. Therefore, the model’s performance on images acquired from other OCT platforms remains uncertain. Transfer learning and device-specific adaptation may be required to extend the model to secondary hospitals or resource-limited regions. In addition, patients with ocular comorbidities were excluded from this study. While this strategy improves internal data consistency, it reduces the representativeness of real-world clinical scenarios, where cataracts frequently coexist with other ocular conditions and may therefore limit the robustness of the model in more complex cases. Although intra-observer variability for the senior ophthalmologist showed good reproducibility, comparison across multiple observers is still needed for a more comprehensive evaluation of human grading variability. Furthermore, the pronounced class imbalance in PSC severity grades may limit the model’s generalizability to populations with different distributions of cataract severity. Among certain patients where a higher grade of PSC may be more prevalent, the model’s performance could differ from that reported here. Adopting calibrated weighted loss functions and exploring hybrid transformer-based architectures may also help address such class imbalances and improve performance under imbalanced conditions in future work. Future studies should focus on multi-center data collection, multi-device validation, and the inclusion of more diverse patient populations to further enhance the robustness and clinical applicability of the proposed model.

## 5. Conclusions

We have presented a comprehensive AI-based solution capable of performing full-region segmentation and grading for all three cataract subtypes on AS-OCT images. The approach achieves competitive grading performance consistent with that of experienced ophthalmologists and demonstrates feasibility for AS-OCT-based PSC assessment. The MMNet demonstrates potential for clinical integration, though external validation is needed before deployment, and may contribute to more standardized cataract assessment across diverse healthcare settings. Further studies are needed to improve the performance of comprehensive automated cataract grading.

## Figures and Tables

**Table 1:** Methodological comparison of previous studies on cataract grading.

Study	Imaging Modality	Cataract Subtypes	Segmentation	Grading Reference Standard	Multi-Task Architecture
Lu et al. 2022 [13]	Slit-lamp	CC/NC/PSC	×	LOCS III	×
Son et al. 2022 [11]	Slit-lamp	CC/NC/PSC	×	LOCS III	×
Gu et al. 2024 [16]	AS-OCT	NC	√	LOCS III	×
Panthier et al. 2025 [12]	SS-OCT	Specified CC/NC/PSC	√	LOCS III	×
Goh et al. 2025 [9]	Slit-lamp/retinal image	CC/NC/PSC	×	Wisconsin system/VSC	×
Han et al. 2025 [17]	AS-OCT	CC/NC	√	LOCS III	×
Takinami et al. 2026 [18]	AS-OCT	CC/NC/PSC	×	LOCS III	×
Our study	AS-OCT	CC/NC/PSC	√	LOCS III	√

CC = cortical cataract; NC = nuclear cataract; PSC = posterior subcapsular cataract; AS-OCT = anterior segment optical coherence tomography; SS-OCT = swept-source optical coherence tomography; Wisconsin system = Wisconsin cataract grading system; VSC = visually significant cataract; LOCS III = lens opacities classification system III.

**Table 2:** Summary of data distribution of AI modeling.

	Cataract Grade						
Category	1	2	3	4	5	6	Total
Cortical							
Training	110	407	689	256	65	-	1527
Validation	28	74	122	35	13	-	272
Test	31	89	126	53	19	-	318
Nuclear							
Training	201	493	260	199	218	156	1527
Validation	44	87	53	29	38	21	272
Test	33	93	59	41	39	53	318
Posterior subcapsular							
Training	987	268	154	77	41	-	1527
Validation	177	49	30	6	10	-	272
Test	152	44	28	53	41	-	318

AI = artificial intelligence.

**Table 3:** Demographic and clinical characteristics of the dataset.

	Training (n = 1527)	Validation (n = 272)	Test (n = 318)	*p* Value
Age (Mean ± SD, years)	71.15 ± 10.78	70.82 ± 11.03	71.17 ± 11.18	0.90
Gender (Male, %)	566(50.3)	131(50.8)	147(49.2)	0.92
Cataract type				
CC	2.84 ± 0.93	2.75 ± 0.97	2.81 ± 1.02	0.27
NC	3.14 ± 1.57	2.97 ± 1.53	3.37 ± 1.64	**0.01**
PSC	1.64 ± 1.03	1.61 ± 1.02	2.33 ± 1.51	**<0.001**

CC = cortical cataract; NC = nuclear cataract; PSC = posterior subcapsular cataract. *p* value was determined by one-way ANOVA, Kruskal–Wallis H test, and the Chi-square test. Bold values indicate significant difference between groups.

**Table 4:** Segmentation and grading performance of the proposed MMNet.

	CC	NC	PSC
Grading Performance			
Sensitivity (%)	84.02 ± 1.32	89.86 ± 1.21	81.13 ± 1.25
Specificity (%)	83.52 ± 1.08	89.29 ± 1.14	81.94 ± 1.13
Accuracy (%)	84.73 ± 1.03	89.76 ± 1.31	82.20 ± 1.41
Precision (%)	82.82 ± 1.13	88.39 ± 1.29	79.57 ± 1.17
F1 Score (%)	83.66 ± 1.21	89.27 ± 1.38	79.49 ± 1.53
Re1.0 (%)	85.53	89.94	83.02
QWK: manual—AI model	0.87	0.95	0.89
Segmentation Performance			
PA (%)	91.71 ± 1.01	95.32 ± 0.77	82.34 ± 1.23
IoU (%)	85.42 ± 1.03	91.98 ± 0.92	60.84 ± 2.14
HD (mm)	9.12 ± 0.34	8.23 ± 0.41	13.32 ± 0.62

CC = cortical cataract; NC = nuclear cataract; PSC = posterior subcapsular cataract; AI = artificial intelligence; QWK = quadratic-weighted kappa; Re1.0 = the percentage of cataract grading absolute prediction errors < 1.0; PA = Pixel Accuracy; IoU = Intersection-over-Union; HD = Hausdorff Distance; accuracy = (true-positive + true-negative)/(true-positive + true-negative + false-positive + false-negative); sensitivity = true-positive/(true-positive + false-negative); Specificity = true-negative/(true-negative + false-positive); precision = true-positive/(true-positive + false-positive); F1 score = 2 × (Precision × Sensitivity)/(Precision + Sensitivity).

**Table 5:** Performance comparison of MMNet, ConvNeXt, and ViT.

Model		ACC (%)	SEN (%)	SPE (%)	F1 Score (%)
ConvNeXt	CC	79.61 ± 2.20	79.75 ± 2.79	94.57 ± 0.60	78.29 ± 2.68
	NC	82.77 ± 2.06	82.69 ± 2.20	96.55 ± 0.41	81.68 ± 2.23
	PSC	77.64 ± 2.33	77.71 ± 2.72	94.34 ± 0.61	74.34 ± 2.72
ViT	CC	80.54 ± 2.17	80.50 ± 2.78	94.85 ± 0.59	78.97 ± 2.66
	NC	84.99 ± 1.95	84.99 ± 2.07	96.98 ± 0.39	84.12 ± 2.10
	PSC	79.52 ± 2.27	79.41 ± 2.69	94.82 ± 0.60	76.20 ± 2.69
MMNet	CC	84.73 ± 1.03	84.02 ± 1.32	83.52 ± 1.08	83.66 ± 1.21
	NC	89.76 ± 1.31	89.86 ± 1.21	89.29 ± 1.14	89.27 ± 1.38
	PSC	82.20 ± 1.41	81.13 ± 1.25	81.94 ± 1.13	79.49 ± 1.53

ACC = accuracy; SEN = sensitivity; SPE = specificity.

**Table 6:** Statistics for the grading performance between doctors and MMNet in the test dataset.

		Senior Doctor	MMNet	Ground Truth	*p* Value
Grade	CC	2.87 ± 1.01	2.88 ± 1.02	2.81 ± 1.02	0.65 ^a^
	NC	3.41 ± 1.61	3.39 ± 1.64	3.37 ± 1.64	0.97 ^a^
	PSC	2.34 ± 1.46	2.40 ± 1.45	2.33 ± 1.51	0.81 ^a^
MAE	CC	0.09 ± 0.29	0.14 ± 0.35	-	**0.04** ^b^
	NC	0.08 ± 0.27	0.10 ± 0.30	-	0.41 ^b^
	PSC	0.10 ± 0.30	0.17 ± 0.38	-	**0.01** ^b^
MSE	CC	0.09	0.14	-	-
	NC	0.08	0.10	-	-
	PSC	0.10	0.17	-	-
Average Grading Time (second per image)		8.89	0.0178	-	-

MAE = mean absolute error; MSE = mean squared error; CC = cortical cataract; NC = nuclear cataract; PSC = posterior subcapsular cataract; *p* value was determined by one-way ANOVA in grading and was determined by Mann–Whitney U test in MAE comparison; bold values indicate significant difference between groups. ^a^: *p* value was determined based on the grade comparison of the senior doctor, MMNet, and ground truth; ^b^: *p* value was determined based on the MAE comparison of the senior doctor, and MMNet in CC, NC, and PSC, respectively.

## Data Availability

The dataset generated during and/or analyzed in the current study is available from the corresponding author on reasonable request.

## Supplementary Materials

The following supporting information can be downloaded at: https://www.mdpi.com/article/10.3390/jcm15072798/s1, Table S1. Summary of age distribution of AI modeling.

## Appendix A. The Detailed Architecture of Artificial Intelligence Modeling

We developed the MMNet, an end-to-end multi-task deep learning framework capable of simultaneously segmenting lens substructures and grading multi-subtype cataract severity from AS-OCT images. The overall pipeline is designed to mirror the clinical workflow in which anatomical delineation precedes disease evaluation. The MMNet first produces an initial coarse segmentation of the cortex, nucleus, and posterior capsule, then progressively refines ambiguous boundaries and extracts lesion-specific multi-granularity features for downstream grading. The architecture integrates anatomical priors with a hierarchical feature-extraction mechanism to achieve subtype-specific, clinically interpretable severity classification.

### Appendix A.1. High-Level Workflow Integration

Following coarse segmentation, the model applies boundary refinement to enhance anatomical fidelity in regions where opacity transitions are subtle or distributed across adjacent subregions. Segmentation masks must be spatially re-sized before guiding subsequent feature extraction, an operation that inherently blurs fine anatomical details. The MMNet incorporates an additional refinement stage to recover high-frequency structural cues and maintain subregion integrity after scaling. The refined masks are then used to isolate lesion-specific features with high spatial precision, minimizing contamination from neighboring lens compartments. Multi-scale structural information is subsequently fused to account for the morphological heterogeneity across cataract subtypes and severity levels. Finally, three parallel grading branches independently evaluate CCs, NCs, and PSCs. Each branch operates exclusively on features derived from its corresponding anatomical region, enabling subtype-specific severity prediction that aligns with clinician diagnostic logic.

### Appendix A.2. Backbone and Coarse Segmentation

MMNet employs a U-Net backbone to generate an initial coarse segmentation mask of the cortex, nucleus, and posterior capsule. Decoder feature maps at multiple semantic granularities serve as the foundation for subsequent refinement and grading tasks.

### Appendix A.3. Multi-Granularity Expert Fusion

The MGEF module adaptively fuses discriminative features across multiple semantic granularities. This process begins with the construction of a multi-granularity feature bank. At each granularity, soft weighting maps are generated from the corresponding decoder feature maps via a fully connected layer followed by a Softmax activation. Guided by these maps, discriminative responses across spatial positions are aggregated via spatial weighting into compact descriptors that capture the most informative semantic patterns of each granularity. Stochastic dropout is subsequently applied to these descriptors to improve robustness. The compact descriptors from all granularities jointly form a multi-granularity feature bank, capturing both fine-grained features and global semantic context. To leverage information from the feature bank effectively, a set of granularity-specific experts is employed. Each expert retrieves patterns via the cross-attention mechanism from the portion of the feature bank corresponding to its granularity. The outputs from these experts are then fused by a gating unit, implemented with two fully connected layers followed by a Softmax activation, which adaptively assigns weights to each expert’s contribution. This gating mechanism emphasizes the most relevant features while suppressing less useful ones, enabling adaptive multi-granularity feature fusion.

### Appendix A.4. Implicit Boundary Rendering

The fused feature map produced by the MGEF module is subsequently fed to the IBR module to address inaccuracies in boundary regions of the coarse segmentation. Specifically, we first randomly sample a set of candidate points from the fused feature map to ensure broad coverage. Among these candidates, points are ranked by their uncertainty in the coarse segmentation map, measured as the confidence gap between the most-likely and the second-most-likely class. Points with the highest uncertainty are preferentially selected, while the remaining ones are uniformly sampled from locations that were not included in the initial candidate set. This strategy ensures that ambiguous boundary regions are more densely represented while still maintaining balanced sampling across the entire representation.

The sampled point coordinates are then used to index their corresponding feature vectors from the fused feature map. Each vector is concatenated with the point’s learnable Fourier feature positional encoding, which enhances the model’s ability to capture high-frequency signals such as boundary regions, and then fed into a shared lightweight multilayer perceptron (MLP) to produce precise point-wise prediction refinements. The refinement method significantly improves boundary delineation accuracy, yielding a fine-grained segmentation mask.

### Appendix A.5. Laplacian Feature Masking

After obtaining the fine segmentation mask, it is paired with the encoder feature map of the corresponding granularity and fed together into the LFM module to extract lesion-specific features, effectively bridging the segmentation and grading tasks by leveraging anatomical spatial priors. Specifically, the LFM processes the fine-grained mask using a learnable resizer that extends beyond conventional interpolation methods. This module employs differential filtering to extract and adaptively integrate multi-frequency details, thereby compensating for information loss typically incurred during interpolation. A gating unit, functioning as an adaptive selector, then performs weighted aggregation of these differential details, preserving the probabilistic semantics of the mask throughout the interpolation. The resized masks are then multiplied element-wise with the corresponding encoder feature maps, enabling the selective extraction of lesion-specific features relevant to each cataract subtype.

An additional MGEF module further fuses these lesion-specific features before being forwarded to fully connected (FC) layers for final grading. To enable simultaneous grading of all cataract subtypes, three parallel grading subnets are implemented, dedicated to CCs, NCs, and PSCs, respectively. Each subnet consists of four LFM modules to extract lesion-specific features at the corresponding granularities, an MGEF block for multi-granularity feature fusion, and an FC classifier for severity prediction. Through this architectural design, the MMNet integrates optimization of both precise lens substructure segmentation and comprehensive multi-subtype cataract severity grading within a unified end-to-end learning framework.
