# Supplementary material for: Multi-Granularity Mask-Guided Network: An Integrated AI Framework for Region-Level Segmentation and Grading of Cataract Subtypes on AS-OCT Images
Source: J Clin Med. 2026 Apr 7;15(7):2798. doi: 10.3390/jcm15072798 (PMC13073274; doi:10.3390/jcm15072798)
Supplement: Supplementary file 1 [file jcm-15-02798-s001.zip › jcm-4143953-supplementary.pdf]

Table S1. Summary of age distribution of AI modeling.

	**Cataract Grade**					
**Category**	1	2	3	4	5	6
**Cortical**						
**Training**	56.16 ± 7.33	69.46 ± 11.59	72.75 ± 9.32	75.49 ± 8.57	74.14 ± 9.01	-
**Validation**	58.96 ± 11.48	69.26 ± 10.59	72.57 ± 10.03	77.84 ± 7.51	71.27 ± 7.25	-
**Test**	59.11 ± 10.45	69.33 ± 10.37	71.95 ± 9.86	78.10 ± 10.80	74.63 ± 9.04	-
**Nuclear**						
**Training**	58.18 ± 9.82	69.83 ± 8.94	75.46 ± 8.31	76.36 ± 8.65	74.73 ± 10.46	73.29 ± 9.02
**Validation**	56.80 ± 9.31	69.27 ± 8.78	76.11 ± 7.75	77.74 ± 9.08	77.31 ± 7.11	74.75 ± 7.81
**Test**	57.76 ± 8.72	67.70 ± 9.23	74.2 ± 10.07	75.92 ± 11.39	75.14 ± 7.75	75.59 ± 10.88
**Posterior subcapsular**						
**Training**	69.85 ± 11.13	72.36 ± 10.59	74.23 ± 8.22	72.96 ± 9.61	78.23 ± 8.35	-
**Validation**	68.90 ± 10.94	73.79 ± 9.64	73.17 ± 11.91	79.2 ± 7.98	78.89 ± 8.80	-
**Test**	67.98 ± 10.94	72.23 ± 11.95	73.84 ± 10.56	74.87 ± 10.50	75.95 ± 8.83	-

Data are presented as mean ± SD. Age is reported in years.
